# The impact of HBV or HCV infection in a cohort of HIV-infected pregnant women receiving a nevirapine-based antiretroviral regimen in Malawi

**DOI:** 10.1186/1471-2334-14-180

**Published:** 2014-04-04

**Authors:** Mauro Andreotti, Maria Franca Pirillo, Giuseppe Liotta, Haswell Jere, Martin Maulidi, Jean-Baptiste Sagno, Richard Luhanga, Roberta Amici, Maria Grazia Mancini, Elisabetta Gennaro, Maria Cristina Marazzi, Stefano Vella, Marina Giuliano, Leonardo Palombi, Sandro Mancinelli

**Affiliations:** 1 Department of Therapeutic Research and Medicines Evaluation, Istituto Superiore di Sanità, Viale Regina Elena 299, 00161 Rome, Italy; 2 Department of Biomedicine and Prevention, University of Tor Vergata, Via Montpellier 1, 00133 Rome, Italy; 3 DREAM Program, Community of S. Egidio, Piazza Sant’Egidio, 00153 Rome, Italy; 4 DREAM Program, Community of S. Egidio, P.O. Box 30355, Blantyre, Malawi; 5 DREAM Program, Community of S. Egidio, Mthengo Wa Ntengo Hospital, Lilongwe, Lumbadzi, Malawi; 6 LUMSA University, Via Transpontina 21, 00193 Rome, Italy

**Keywords:** Antiretroviral therapy, Hepatotoxicity, HBV, HCV, Nevirapine, Pregnancy, Mother-to-child HIV transmission

## Abstract

**Background:**

Coinfection with the hepatitis viruses is common in the HIV population in sub-Saharan Africa. The aim of this study was to assess, in a cohort of HIV-infected pregnant women receiving antiretroviral drugs (ARVs), the prevalence of HBV and HCV infections and to determine the impact of these infections on the occurrence of liver toxicity and on the viro-immunological response.

**Methods:**

Women were screened for HBsAg and HCV-RNA before starting, at week 25 of gestational age, an antiretroviral regimen consisting of lamivudine and nevirapine plus either stavudine or zidovudine. Women with CD4+ < 350/mm^3^ continued ARVs indefinitely, while the other women interrupted treatment 6 months postpartum (end of breastfeeding period). Both groups were followed for 2 years after delivery. Liver function was monitored by alanine aminotransferase (ALT) measurement. The Cox proportional hazards model was used to identify factors associated with the emergence of liver toxicity.

**Results:**

A total of 28 women out of the 309 enrolled in the study (9.1%) were coinfected with HBV (n. 27), or HCV (n. 1). During follow-up 125 women (40.4%) developed a grade ≥ 1 ALT elevation, 28 (9.1%) a grade ≥ 2 and 6 (1.9%) an elevation defining grade 3 toxicity. In a multivariate model including age, baseline CD4+ count and hemoglobin level, the presence of either HBV or HCV infection was significantly associated with the development of an ALT increase of any grade (P = 0.035). Moderate or severe liver laboratory toxicity (grade ≥ 2) was more frequent among women with baseline CD4+ > 250/mm^3^ (P = 0.030). In HBV-infected women a baseline HBV-DNA level above 10,000 IU/ml was significantly associated to the development of liver toxicity of grade ≥ 1 (P = 0.040). Coinfections had no impact on the immunological and virological response to antiretroviral drugs up to 2 years after delivery.

**Conclusions:**

In this cohort of nevirapine-treated women the presence of HBV or HCV was associated only to the development of mild liver toxicity, while the occurrence of moderate or severe hepatoxicity was correlated to a baseline CD4+ count > 250/mm^3^. No statistically significant effect of the coinfections was observed on the efficacy of antiretroviral therapy.

## Background

Coinfection with the hepatitis B (HBV) or C (HCV) viruses occurs frequently in the HIV-infected population. Prevalence of the coinfections differs depending on the geographical areas: HCV is more prevalent in western countries, while HBV is highly endemic in sub-Saharan Africa and Asia [1]. The impact of these coinfections in the HIV-population in resource-limited countries has not been fully elucidated, although some studies have reported an increase in liver toxicity in the coinfected patients [2-4], and others have shown little influence on the response to antiretroviral treatment [5,6]. Limited information is available on the possible interactions between the presence of hepatitis coinfections and the administration of potentially hepatotoxic drugs including the non-nucleoside reverse transcriptase inhibitor, nevirapine [7]. Data obtained on this topic during pregnancy are particularly relevant, because of the increased risk of drug-related toxicity reported for HIV-infected pregnant women [8].

The aim of this study was to evaluate the role of the coinfection with hepatitis B or C viruses on the development of liver toxicity and on the virological and immunological response in HIV-infected pregnant women receiving a nevirapine-based antiretroviral combination regimen, either for prevention of mother-to-infant transmission only, or for treatment of their own health.

## Methods

### Study population

Patient population included women enrolled in the SMAC (Safe Milk for African Children) study, an observational study conducted in Malawi and aimed to assess the safety and pharmacokinetics of ART (Anti Retroviral Therapy) administration to breastfeeding women [9]. The study was conducted within the DREAM (Drug Resources Enhancement against AIDS and Malnutrition) Program, designed and managed by the Community of S. Egidio, an italian faith-based non-governmental organization. The study was approved by the National Health Sciences Research Committee of Malawi (approval number #486). A separate informed consent was signed by all participants. HIV-positive pregnant women, naive to antiretrovirals, older than 16, with no baseline grade 3 or 4 laboratory toxicity and no concomitant treatment for tuberculosis were eligible for the study. ART was administered as soon as possible after the first trimester if the women met the criteria for treatment (CD4+ < 350/mm^3^), and treatment was continued indefinitely after pregnancy. The women not needing treatment for themselves received antiretroviral prophylaxis from week 25 of gestational age until 6 months after delivery, which represented the recommended duration of breastfeeding at the time of the study (enrolment occurred between February 2008 and February 2009). All women received a combination regimen based on lamivudine (3TC, 150 mg twice daily) and nevirapine (NVP, 200 mg twice daily) plus either stavudine (d4T, 30 mg twice daily) or zidovudine (ZDV, 300 mg twice daily). Women experiencing nevirapine-associated toxicity were allowed to substitute this drug with lopinavir-ritonavir (LPV/r, 400/100 twice daily). Women were followed for 2 years after delivery. Complete blood count (Sysmex KX-21N hematology analyzer, Sysmex Corporation, Kobe, Japan) and liver enzymes (Humastar 180 Chemistry Analyzer, Human Diagnostics, Germany) were determined every 2 weeks for the first 2 months and every month thereafter during pregnancy, at 1, 3 and 6 months postpartum and then every three months until 24 months. CD4+ cell count (Epics XL-MCL, Beckman Coulter, Brea, CA, USA) and HIV-RNA determination (branched-DNA version 3.0 assay, Siemens Diagnostics, Deerfield, IL, USA) were performed every 6 months after delivery. Laboratory abnormalities were classified according to the Division of Acquired Immunodeficiency Syndrome/National Institute of Allergy and Infectious Diseases (DAIDS/NIAID) grading system, 2004 revision [10]. An ALT level of 1.25-2.5 times the upper limit of normal (ULN) defined grade 1 hepatoxicity, 2.6-5 times the ULN defined grade 2, 5.1-10 times the ULN defined grade 3, and > 10 times the ULN defined grade 4 hepatoxicity.

### Laboratory procedures

For HBV, women were tested using the Murex HBsAg Version 3 with Confirmatory Assay (Murex Biotech, Dartford, UK). The presence of HBeAg was assessed using the ARCHITECT HBeAg Reagent Kit (Abbott Diagnostics Division, Wiesbaden, Germany). Serum HBV-DNA was quantified using the COBAS TaqMan HBV Test (Roche Molecular System, Branchburg, NJ, USA) with a limit of quantification of 6 IU/ml. For HCV all sera were first tested with the Innotest HCV Ab IV assay (Innogenetics, Ghent, Belgium). Low and high-positive results were re-assayed using the Ortho HCV 3.0 ELISA test (Ortho-Clinical Diagnostics, Raritan, NJ, USA). Samples reactive with both assays were considered anti-HCV positive [11]. In anti-HCV positive women the presence of HCV-RNA was tested in at least 3 different samples using the Versant HCV-RNA 1.0 assay (Siemens Diagnostics) with a detection limit of 15 IU/ml. Only patients with detectable HCV-RNA were considered HCV-positive.

### Data analysis

Characteristics in the hepatitis coinfected and the HIV monoinfected groups were compared using the Mann–Whitney test for continuous variables and the chi square or Fisher’ s exact test for categorical variables. The Kaplan-Meier method was used to define the time to the development of liver toxicity and the Cox proportional hazards model to identify factors associated with its emergence. Variables were included in the multivariate model if they were significant at a P value of less than 0.25 in univariate analysis. For all statistical tests, two-sided P values less than 0.05 were considered to be significant. Data analysis was done using the SPSS software system 20.0 (IBM, Somers, NY, USA).

## Results

A total of 309 HIV-infected women out of the total 311 enrolled in the study were tested for HBV and HCV at baseline (before drug administration). Twenty-seven women (8.7%) were HBsAg + and 1 (0.3%) was HCV-RNA positive (8 women were positive only for anti-HCV antibodies). Therefore, a total of 28 women out of 309 (9.1%) were coinfected with either HBV or HCV virus. Among the 27 HBV-infected women, 10 (37%) were HBeAg + and 18 out of the 24 tested (75%) had detectable (> 6 IU/ml) HBV-DNA at baseline. Median HBV-DNA among those with detectable DNA was 1,231 IU/ml (IQR 37–38,500,000). Eight out of 24 (33.3%) had > 10,000 IU/ml, and all of them were HBeAg-positive. Six of the 16 (37.5%) HBeAg-negative women tested for HBV-DNA had un undetectable HBV viral load (< 6 IU/ml), and the others had levels ≤ 2,000 IU/ml.

Eight women were lost to follow-up before delivery, 3 had a miscarriage and left the study and 48 (15.5%) were lost between delivery and Month 24. A total of 147 women (including 13 with coinfection) interrupted treatment 6 months postpartum when the risk of breastfeeding transmission was ceased, while the remaining 162 (including 15 with coinfection), who had baseline CD4+ < 350/mm^3^, continued antiretrovirals indefinitely. Women were followed for a median of 111 weeks (IQR: 105–116). Patient characteristics are reported in Table 1. At baseline only 2 monoinfected women had a grade 1 ALT elevation; coinfected women had a higher (although not clinically relevant) ALT level compared to the HIV + only group (P = 0.043). No other significant difference was observed at baseline between the two groups.

**Table 1:** Patient characteristics

	**HBV or HCV coinfected (n.28)**	**HIV + only (n. 281)**	**P value**
Age			
Median (IQR)	27.5 (23–30)	27 (23–31)	0.960
WHO Stage N. (%)			
I	17 (63)	211 (75.9)	0.493
II	7 (25.9)	47 (16.9)	
III	3 (11.1)	19 (6.8)	
IV		1 (0.4)	
Week of gestation at ART initiation			
Median (IQR)	27 (24–31)	26 (24–30)	0.576
Duration of ART during pregnancy			
Median days (IQR)	78 (49–92)	70 (44–95)	0.828
ART regimen, N. (%)			
Stavudine/Lamivudine/Nevirapine	15 (53.6)	148 (52.7)	
Zidovudine/Lamivudine/Nevirapine	13 (46.4)	133 (47.3)	0.927
Baseline Hemoglobin (g/dl)			
Median (IQR)	9.8 (9.0-10.9)	10.3 (9.5-11.3)	0.082
Baseline Platelets (x10^3^/mm^3^)			
Median (IQR)	177 (126–262)	202 (168–249)	0.106
Baseline ALT (IU/ml)			
Median (IQR)	15.5 (12.2-27.1)	12.5 (10–17.8)	0.043
Baseline AST (IU/ml)			
Median (IQR)	28 (23.3-37.1)	25.1 (19.3-34.2)	0.209
Baseline CD4+ cell count/mm^3^			
Median (IQR)	327 (193–495)	340 (219–494)	0.666
Baseline HIV-RNA log_10_/ml			
Median (IQR)	3.89 (3.0-4.5)	4.09 (3.4-4.6)	0.297

### Hepatoxicity

**Figure 1:**
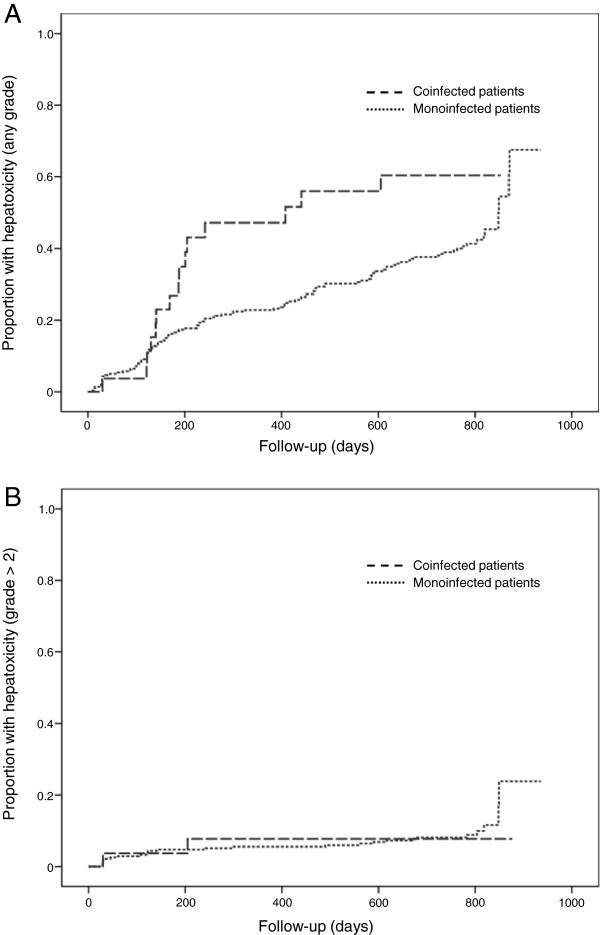
Proportion of patients who experienced episodes of hepatoxicity of any grade (Panel A) or hepatoxicity of grade ≥ 2 (Panel B) by infection group.

Over the total follow-up time 125 women (40.5%) developed a new grade ≥ 1 ALT elevation (15/28 or 53.6% coinfected women and 110/281 or 39.1% HIV + only), 28 (9.1%) (2 coinfected and 26 HIV + only) a grade ≥ 2, and 6 (1.9%) (all HIV + only) a grade 3 toxicity. Twelve % of the cases occurred during pregnancy and 57.6% within 6 months of delivery (83.3% of the cases in coinfected women and 53.2% of the cases occurring in monoinfected women). In a Kaplan-Meier univariate analysis, the risk of developing an ALT elevation of any grade was higher for HBV or HCV infected women (P = 0.033 by log-rank test), while no significant association was found for grade ≥ 2 liver toxicity (P = 0.706) (Figure 1). In a multivariate Cox model that adjusted for age, CD4+ count > or < 250/mm^3^ and hemoglobin level, the presence of hepatitis B or C remained significantly associated with the development of liver toxicity of any grade (hazard ratio [HR]: 1.797, 95% CI 1.042-3.098, P = 0.035) (Table 2). In a similar multivariate model, based on the same variables reported above, but that considered only grade ≥ 2 ALT elevations, the only predictor of the emergence of toxicity was a baseline CD4+ cell count > 250/mm^3^ (HR: 3.296, 95% CI 1.125-9.655, P = 0.030).

**Table 2:** Cox proportional hazards analysis of factors associated with the emergence of hepatotoxicity of any grade

**Variable**	**Univariate analysis**		**Multivariable analysis**	
	**Hazard ratio (95% CI)**	**P value**	**Hazard ratio ( 95% CI)**	**P value**
Age (per 1-year increase)	1.021 (0.987-1.057)	0.230	1.025 (0.990-1.061)	0.162
CD4+ cell count (>250/mm^3^ vs ≤ 250/mm^3^)	1.257 (0.853-1.851)	0.247	1.397 (0.940-2.075)	0.098
Hemoglobin (<median level of 10.3 g/dl vs ≥ median)	1.275 (0.897-1.811)	0.176	1.320 (0.924-1.886)	0.128
Presence of HBV or HCV infection	1.788 (1.041-3.073)	0.035	1.797 (1.042-3.098)	0.035

Fifteen cases of ALT increase of any grade and 8 cases of grade ≥ 2 increase developed during pregnancy. Thirteen out of the 28 (46.4%) total cases of grade ≥ 2 toxicity developed within the first 18 weeks of treatment. Of these cases, 12 occurred in the 211 women with baseline CD4+ > 250/mm^3^ and 1 among the 98 women with baseline (CD4+ ≤ 250/mm^3^, P = 0.069). There was no difference in the occurrence of toxicity after 18 weeks according to the baseline CD4+ stratum.

In HBV-infected women, after controlling for age, CD4+ count above or below 250/mm^3^ and hemoglobin level, a baseline HBV-DNA level > 10,000 IU/ml was significantly associated with the development of liver toxicity of any grade (HR:3.278, 95% CI 1.056-10.180, P = 0.040). HBV-DNA measurements during treatment were available for 20 women at Month 6 and for 14 women at Month 12 postpartum. At 6 months 9/20 had a detectable HBV-DNA level (but only 3 had levels > 10,000 IU/ml) while at 12 months 8/14 had detectable HBV-DNA levels (5 had levels above 10,000 IU/ml), but it has to be considered that 6 of these women, not meeting the criteria for treatment, had interrupted treatment 6 months postpartum. Women with detectable HBV-DNA at 6 months had a higher median ALT level at 12 and 24 months compared to those with undetectable HBV-DNA (59.2 vs 18.5 IU/ml and 35.9 vs 18.2 IU/ml, respectively, P = 0.001 and P = 0.043).

There was no difference in the probability of developing liver toxicity of any grade between women receiving zidovudine or stavudine.

Among the 3 women who discontinued nevirapine because of liver toxicity none was coinfected.

**Figure 2:**
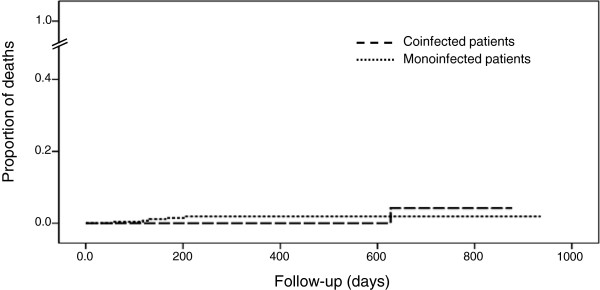
Proportion of deaths by infection group.

Among the 6 women who died during follow-up, one was coinfected with HBV (no death was liver-related). There was no difference in the probability of survival between the 2 groups (P = 0.511 by log-rank test) (Figure 2).

### Viro-immunological response

**Figure 3:**
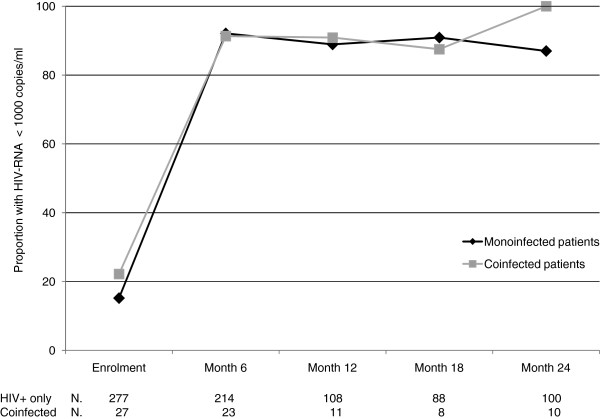
**Proportion of women with HIV-RNA < 1,000 copies/ml during follow-up.** After 6 months only women on treatment were included in the analysis.

Among the 237 women with HIV-RNA determination 6 months postpartum, 21/23 coinfected women vs 160/214 HIV + only had < 50 HIV-RNA copies/ml (P = 0.118). No difference was detected also considering the proportion of women with HIV-RNA < 1,000 copies/ml (21/23 vs 197/214, P = 1.0). Among the women continuing therapy, the proportion of women with < 50 copies/ml or < 1,000 copies/ml was not significantly different between the coinfected and the monoinfected populations at Month 12 or 24 (Figure 3).

**Figure 4:**
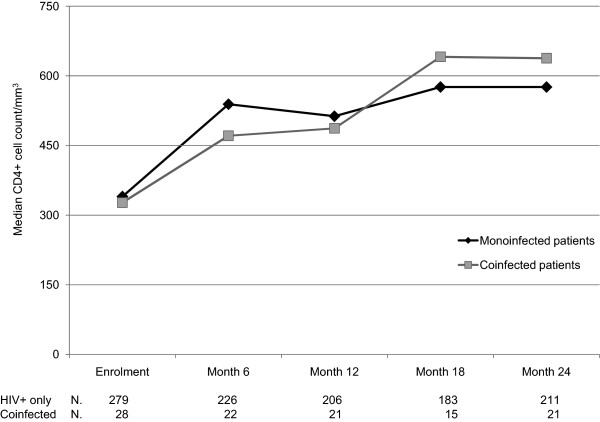
CD4+ cell counts during follow-up by infection group.

Median CD4+ increase at Month 6 was 185 cells/mm^3^, with no difference between the two groups (185 cells in the monoinfected vs 179 cells in the coinfected group, P = 0.819) (Figure 4). Among the women continuing therapy, the median CD4+ increases at Month 12 and Month 24 were 218 and 294 cells, respectively with no difference between those coinfected and those HIV + only (279 vs 217 cells, and 439 vs 294 cells, respectively, P = 0.123 and P = 0.172).

## Discussion

Emergence of severe liver toxicity in this cohort of women was infrequent. Women with either coinfection had a higher risk of mild hepatoxicity, mainly occurring in the first 6 months of treatment, and among the HBV-positive women baseline levels of HBV-DNA above 10,000 IU/ml were significantly associated with the emergence of toxicity. However, it has to be underlined that a significant association was found only for grade ≥ 1 ALT increases, while no effect was found for the occurrence of moderate to severe liver toxicity, suggesting an overall impact of modest magnitude.

More frequent ALT elevations were observed in coinfected patients in other reports [2-4,6,7] and HBV-DNA was found to be associated with liver toxicity in some studies [5] but not in others [6]. Our finding of a correlation between detectable levels of HBV-DNA during follow-up and values of ALT supports the role of actively replicating HBV infection in the occurrence of liver toxicity. In our study both incomplete response to therapy and interruption of treatment 6 months postpartum for women not meeting the criteria for treatment, could be responsible for high HBV-DNA levels during follow-up. At this regard, recent World Health Organization guidelines for the prevention of vertical transmission of HIV [12], recommending life-long antiretroviral therapy containing two anti-HBV active agents for all pregnant women, represent an important step to minimize the risk of hepatic flares in coinfected patients.

High levels of CD4+ are known to be associated with nevirapine-induced liver toxicity [13], and in our cohort of nevirapine-treated women baseline levels of CD4+ > 250/mm^3^ were indeed correlated to the emergence of grade ≥ 2 ALT elevation. Liver toxicity associated with nevirapine is supposed to occur more frequently in the first 18 weeks of treatment, and in our study grade ≥ 2 early toxicity occurred almost exclusively among women with baseline CD4+ count > 250/mm^3^. However, the overall incidence of moderate or severe liver toxicity (9%) was relatively low, considering the long-term follow-up (and less than 1% of the women discontinued nevirapine because of liver toxicity), thus confirming our previous observation of a relative safety of using nevirapine in this context [14]. Also, only 2.5% of the women in our study had grade ≥ 2 events during pregnancy in line with the findings of a recent meta-analysis [15], indicating that drug adverse events were not greater in pregnant women receiving nevirapine than in the general population. The findings of a study performed in Cameroon [7], in patients with CD4+ < 350/mm^3^, have also suggested that a nevirapine-based antiretroviral regimen could be used safely as first-line therapy despite frequent coinfections with the hepatitis viruses.

Previous studies have evaluated the impact of HBV or HCV on the response to ART in resource-limited settings. Although a limited impact of coinfections has been shown on the antiretroviral response, some studies [4,16] have shown a negative effect on the CD4+ response in HBV-coinfected patients, especially in those with HBe or high HBV-DNA levels, and some a slower virological response [6]. The low numbers in our study limited the power to detect significant differences in the viro-immunological parameters however, we did not observe any negative trend in terms of long-term virological suppression and immunological response.

Our study also confirmed that in Malawi HBV infection is highly prevalent in the HIV-positive population [17] with more than 8% of the HIV-infected women positive for HBsAg in our cohort, and that HCV is quite rare, in line with other findings [18]. Among the HBV-infected women, HBe antigen was present in about one third of patients, in agreement with previous reports [16], while the proportion of those with > 10,000 IU/ml of HBV-DNA was low compared to other studies [3,16]. A role of different genotypes can be hypothesized since genotypes C, D and F are associated to higher DNA levels compared to other genotypes [3] and in Malawi genotype A is highly dominant [19].

Limitations of this study include: the small number of coinfected women in the cohort that often did not allow to draw statistically significant conclusions; the lack of treatment adherence measures to determine the possible impact of unscheduled suspension of drugs; and incomplete data on the HBV-DNA levels during follow-up in the HBV-infected women, although the available data support the role of actively replicating infection in determining liver enzymes elevations.

## Conclusions

In this cohort of HIV-infected nevirapine-treated pregnant women in Malawi the presence of HBV or HCV infection seemed not to have a major impact on the emergence of moderate to severe liver toxicity and on the response to antiretroviral treatment up to 24 months after delivery. High baseline levels of HBV-DNA were significantly associated with the emergence of mild liver toxicity. Screening for the hepatitis viruses in these settings could be useful to identify the women that could benefit from a closer monitoring strategy.

## Abbreviation

HBV: Hepatitis B virus; HCV: Hepatitis C virus; HBsAg: Hepatitis B surface antigen.

## Competing interests

Stefano Vella has received honoraria from ViiV for consultancy, and from Gilead and Janssen for board membership. All other authors have no conflict to declare.

## Authors’ contributions

MA participated in the design and the conduction the study and contributed in drafting the paper, MP designed and supervised the laboratory procedures, GL was responsible for data collection and management HJ, MM and JBS were responsible for the clinical care of the enrolled patients, RL, RA, MGM, and EG were involved in the laboratory assays and in data analysis, MCM and SV participated in the design of the study and critically revised the manuscript, MG participated in the design of the study, performed the statistical analysis and drafted the manuscript, LP conceived the study and participated in its design and coordination, SM was responsible for study coordination and data interpretation. All authors read and approved the final manuscript.

## Pre-publication history

The pre-publication history for this paper can be accessed here:

http://www.biomedcentral.com/1471-2334/14/180/prepub
